# Lost Small Envelope Protein Expression from Naturally Occurring PreS1 Deletion Mutants of Hepatitis B Virus Is Often Accompanied by Increased HBx and Core Protein Expression as Well as Genome Replication

**DOI:** 10.1128/JVI.00660-21

**Published:** 2021-06-24

**Authors:** Shuwen Fu, Jing Zhang, Quan Yuan, Qianru Wang, Qiang Deng, Jisu Li, Ningshao Xia, Yongxiang Wang, Yumei Wen, Shuping Tong

**Affiliations:** a Department of Pathobiology, Key Laboratory of Medical Molecular Virology, School of Basic Medical Sciences, Fudan University, Shanghai, China; b State Key Laboratory of Molecular Vaccinology and Molecular Diagnostics, National Institute of Diagnostics and Vaccine Development in Infectious Diseases, School of Public Health, Xiamen University, Xiamen, China; c Liver Research Center, Rhode Island Hospital and Warren Alpert Medical School of Brown University, Providence, Rhode Island, USA; University of Southern California

**Keywords:** hepatitis B virus, preS1 deletion, envelope proteins, hepatitis B surface antigen, HBx protein, genome replication, transcriptional interference

## Abstract

Hepatitis B virus (HBV) transcribes coterminal mRNAs of 0.7 to 3.5 kb from the 3.2-kb covalently closed circular DNA, with the 2.1-kb RNA being most abundant. The 0.7-kb RNA produces HBx protein, a transcriptional transactivator, while the 3.5-kb pregenomic RNA (pgRNA) drives core and P protein translation as well as genome replication. The large (L) and small (S) envelope proteins are translated from the 2.4-kb and 2.1-kb RNAs, respectively, with the majority of the S protein being secreted as noninfectious subviral particles and detected as hepatitis B surface antigen (HBsAg). pgRNA transcription could inhibit transcription of subgenomic RNAs. The present study characterized naturally occurring in-frame deletions in the 3′ preS1 region, which not only codes for L protein but also serves as the promoter for 2.1-kb RNA. The human hepatoma cell line Huh7 was transiently transfected with subgenomic expression constructs for envelope (and HBx) proteins, dimeric constructs, or constructs mimicking covalently closed circular DNA. The results confirmed lost 2.1-kb RNA transcription and HBsAg production from many deletion mutants, accompanied by increases in other (especially 2.4-kb) RNAs, intracellular HBx and core proteins, and replicative DNA but impaired virion and L protein secretion. The highest intracellular L protein levels were achieved by mutants that had residual S protein expression or retained the matrix domain in L protein. Site-directed mutagenesis of a high replicating deletion mutant suggested that increased HBx protein expression and blocked virion secretion both contributed to the high replication phenotype. Our findings could help explain why such deletions are selected at a late stage of chronic HBV infection and how they contribute to viral pathogenesis.

**IMPORTANCE** Expression of hepatitis B e antigen (HBeAg) and overproduction of HBsAg by wild-type HBV are implicated in the induction of immune tolerance to achieve chronic infection. How HBV survives the subsequent immune clearance phase remains incompletely understood. Our previous characterization of core promoter mutations to reduce HBeAg production revealed the ability of the 3.5-kb pgRNA to diminish transcription of coterminal RNAs of 2.4 kb, 2.1 kb, and 0.7 kb. The later stage of chronic HBV infection often selects for in-frame deletions in the preS region. Here, we found that many 3′ preS1 deletions prevented transcription of the 2.1-kb RNA for HBsAg production, which was often accompanied by increases in intracellular 3.5-, 0.7-, and especially 2.4-kb RNAs, HBx and core proteins, and replicative DNA but lost virion secretion. These findings established the biological consequences of preS1 deletions, thus shedding light on why they are selected and how they contribute to hepatocarcinogenesis.

## INTRODUCTION

**FIG 1:**
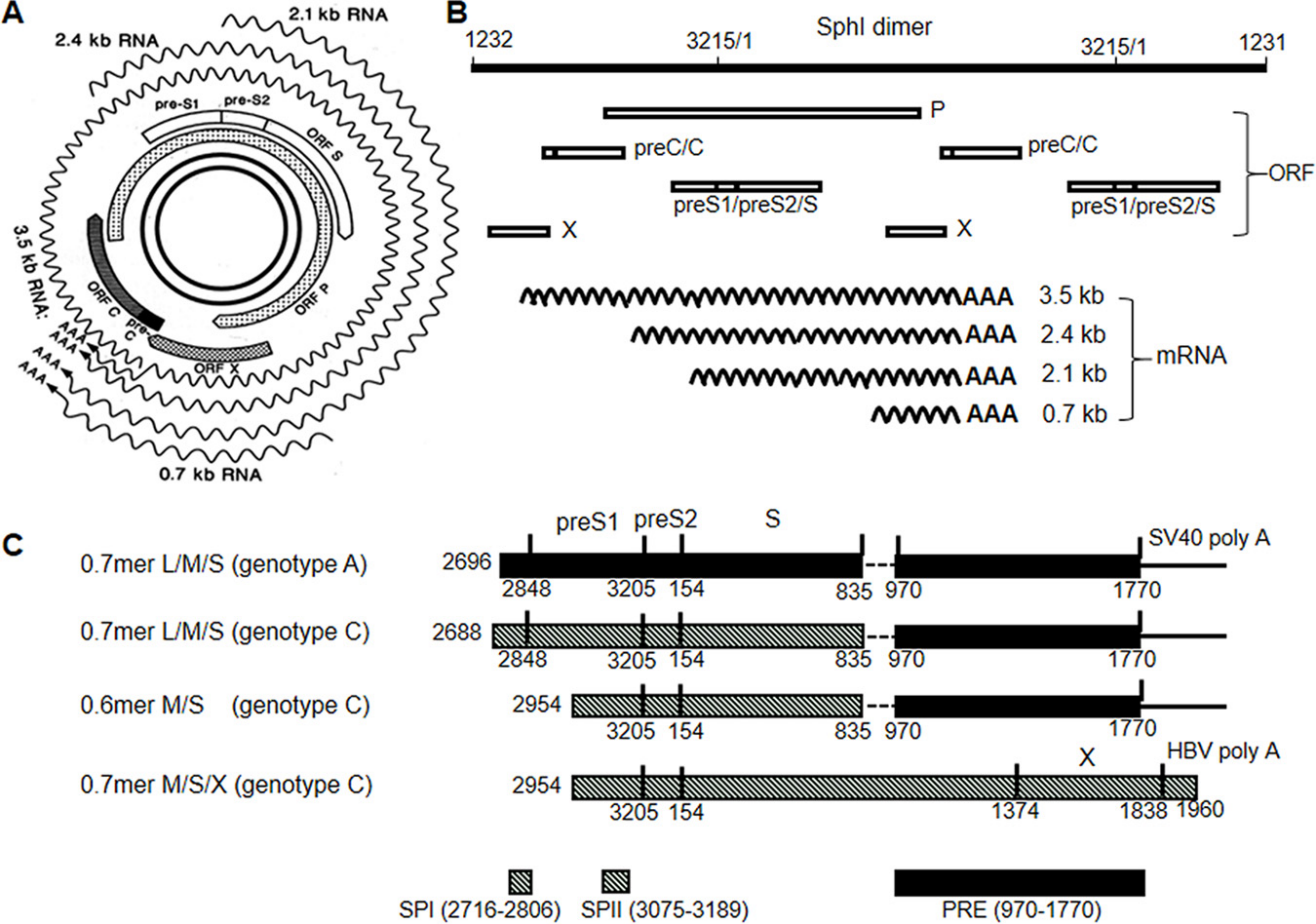
Schematic view of HBV RNA transcription, envelope protein expression, and genome replication from cccDNA, SphI dimer, and subgenomic expression constructs. (A) From the inside out are the double-stranded HBV genome as cccDNA, four overlapping genes or open reading frames (ORFs), and four sizes of coterminal RNAs (wavy lines). Please note that the 5′ end of the 3.5-kb RNA overlaps the 3′ end of all subgenomic RNAs. (B) The SphI dimer of genotype C as a functional equivalent of cccDNA. The top line is two tandem copies of the 3,215-nt HBV genome joined by the unique SphI site (not shown). Below are the four ORFs, and at the bottom are four sizes of HBV RNAs (wavy lines). Here, the transcription unit for 2.1-kb RNA lies downstream of those for 3.5- and 2.4-kb RNAs but upstream of that for 0.7-kb RNA. (C) Subgenomic expression constructs for HBV envelope proteins. The 0.7-mer L/M/S construct and the 0.6-mer M/S construct of geno27.2 of genotype C were derived from the 0.7-mer L/M/S construct of genotype A by replacement of the 5′ end of the HBV sequence. There is a gap of 136 nt in the HBV sequence, and the X gene (nucleotides 1771 to 1838) is incomplete at the 3′ end. Also, the poly(A) signal is derived from SV40. The 0.7-mer M/S/X construct of geno27.2 was generated by inserting a continuous HBV DNA fragment into the pBluescript SK(−) vector. It has an intact X gene and the poly(A) signal from HBV (nucleotides 1916 to 1921). The locations of the SPI and SPII promoters and the posttranscriptional regulatory element (PRE) are indicated.

Hepatocellular carcinoma (HCC) is the sixth most prevalent cancer and the third most common cause of cancer deaths. More than 60% of HCCs are caused by chronic infection with hepatitis B virus (HBV) (1, 2). HBV is an enveloped virus with a 3.2-kb partially double-stranded DNA genome, which harbors four genes, i.e., precore/core, envelope (preS/S), polymerase (P), and X (3). All four genes are located on the plus-strand DNA and cover the entire circular genome, with the P gene overlapping entirely with the envelope gene and partially with the core and X genes (Fig. 1A). Both the precore/core and envelope genes can produce related proteins through alternative translation initiation from in-frame ATG codons. Thus, the large (L), middle (M), and small (S) envelope proteins are products of translation initiation from the preS1, preS2, and S regions of the envelope gene, respectively. L protein has an extra preS1 domain (of 119 amino acids [aa] for most HBV genotypes) in comparison with M protein, which has an extra preS2 domain of 55 aa in comparison with S protein of 226 aa. The core gene encodes the core protein, the building block of the capsid (core particle). Translation initiated from the precore region generates fused precore/core protein, which is converted by proteolytic cleavage into hepatitis B e antigen (HBeAg), a secreted and nonparticulate variant of core protein involved in induction of immune tolerance (4, 5).

HBV protein expression and genome replication are driven by covalently closed circular DNA (cccDNA) in the nucleus of infected hepatocytes, which is initially converted from relaxed circular DNA of incoming virions. Four forms of mRNAs, of 3.5, 2.4, 2.1, and 0.7 kb, are transcribed from the cccDNA template through core, SPI, SPII, and X promoters, respectively, further augmented by two enhancer elements. All of these RNAs are unidirectional and coterminal (Fig. 1A), thus raising the possibility of transcriptional interference (6–8). The 2.4-kb and 0.7-kb RNAs serve as mRNAs for L protein and HBx protein, respectively, the translation product of the X gene and a transcriptional transactivator (9). Both the 3.5-kb and 2.1-kb RNAs have heterogeneous 5′ ends straddling in-frame AUG codons, which enables the 2.1-kb RNA to translate M protein in addition to abundant S protein. The longer 3.5-kb RNA, called precore RNA (pcRNA), is the mRNA for precore/core protein. The shorter one, pregenomic RNA (pgRNA), lacks the 5′ end of the precore region and thus expresses core protein instead. It is also used for P protein expression through ribosomal leaky scanning. The 5′ end of pgRNA forms a stem-loop structure for its encapsidation into core particles, where it is converted to double-stranded DNA by a series of enzymatic reactions catalyzed by the copackaged P protein. Therefore, the pgRNA alone is sufficient to drive HBV genome replication. Subsequent virion formation initiates with core particle interaction with the matrix domain on L protein (10, 11). L protein further retains the S protein for its incorporation into enveloped core particles (virions), with particle secretion driven by the S protein. As the most abundant envelope protein, the S protein is mostly secreted with M protein as 22-nm subviral particles (SVPs) lacking internal capsids (12, 13). In fact, SVPs exceed the 42-nm virions by 1,000- to 100,000-fold. The abundant S domain on SVPs and virions, which is detected as hepatitis B surface antigen (HBsAg), serves as a sensitive serological marker of ongoing HBV infection. Secretion of SVPs is inhibited by L protein, especially at higher L/S protein ratios, whereas optimal virion secretion requires a proper L/S protein ratio (14–17).

HBeAg and SVPs are dispensable for the HBV life cycle at the cellular level but are implicated in HBV persistence in the human host through induction of immune tolerance. Whereas <10% of acute adult HBV infections become chronic, more than 90% of maternal-infant transmissions lead to chronic infection. Such a perinatal mode of transmission is common in East Asia, where HBV genotypes B and C predominate. These chronic carriers are initially positive for both HBeAg and HBsAg, accompanied by high viremia titers but normal liver function. At this stage, the viral genome in the blood is highly homogeneous and is referred to as wild type (WT). Liver injury and hepatitis occur at the subsequent immune clearance (IC) phase, which can drive the sequential selection of adaptive mutations in different parts of the viral genome (18–20). A landmark during the IC phase is HBeAg seroconversion (loss of HBeAg followed by rise of anti-HBe antibody), which is often associated with the emergence of mutations to suppress HBeAg expression. Core promoter mutations (CPMs) reduce HBeAg production at the transcriptional level, while precore mutations (PCMs) abolish HBeAg expression at the translational level by premature translational termination. The most common PCM is the G1896A nonsense mutation, while the A1762T/G1764A double mutation is the most common CPM. These mutations are relatively simple, and their functional consequences have been extensively characterized (21–27). The later stage of the IC phase prior to HBsAg seroconversion (loss of HBsAg followed by rise of anti-HBs antibodies) further selects for mutations in the envelope gene. The best known and most characterized are immune escape mutations in the S region, which can lead to vaccine escape (28–33).

Common mutations in the preS region include mutated preS2 ATG codons to prevent M protein expression (34–39) and in-frame preS deletions to truncate the L, M, or both proteins (for a recent review, see reference 40). The latter include short deletions covering the preS1 ATG codon and large deletions removing the central/3′ preS1 region or the 5′/central preS2 region (35, 37–39, 41–51). Some preS deletions are highly prevalent in viremic HCC patients, suggesting their contribution to hepatocarcinogenesis (38, 39, 43, 45, 47, 48, 50, 52–54). The preS deletions are highly heterogeneous, and only limited studies have attempted to characterize their biological consequences (39, 55–59). We recently found that 15-nucleotide (nt) and 18-nt deletions in the 5′ preS1 region of genotype C to shorten the L protein like WT genotype D markedly enhanced HBV infectivity in cell culture (60). Since the 3′ preS1 region overlaps the SPII promoter for transcription of the 2.1-kb RNA (61, 62), some preS1 deletions may impair HBsAg production at the transcriptional level. In previous studies, our detailed characterization of CPMs in genotype A uncovered the ability of the 3.5-kb pgRNA to suppress transcription of subgenomic RNAs (26, 63, 64). The objective of the present study was to characterize the impact of naturally occurring 3′ preS1 deletions on 2.1-kb RNA transcription and S protein expression, as well as HBsAg and virion secretion. Considering that the 2.1-kb RNA is the most abundant HBV transcript for WT virus, we also examined whether loss of the 2.1-kb RNA could increase HBV genome replication and core, HBeAg, L, and HBx protein expression due to increased transcription of corresponding mRNAs.

## RESULTS

### Naturally occurring and artificial preS deletion mutants for functional characterization.

**FIG 2:**
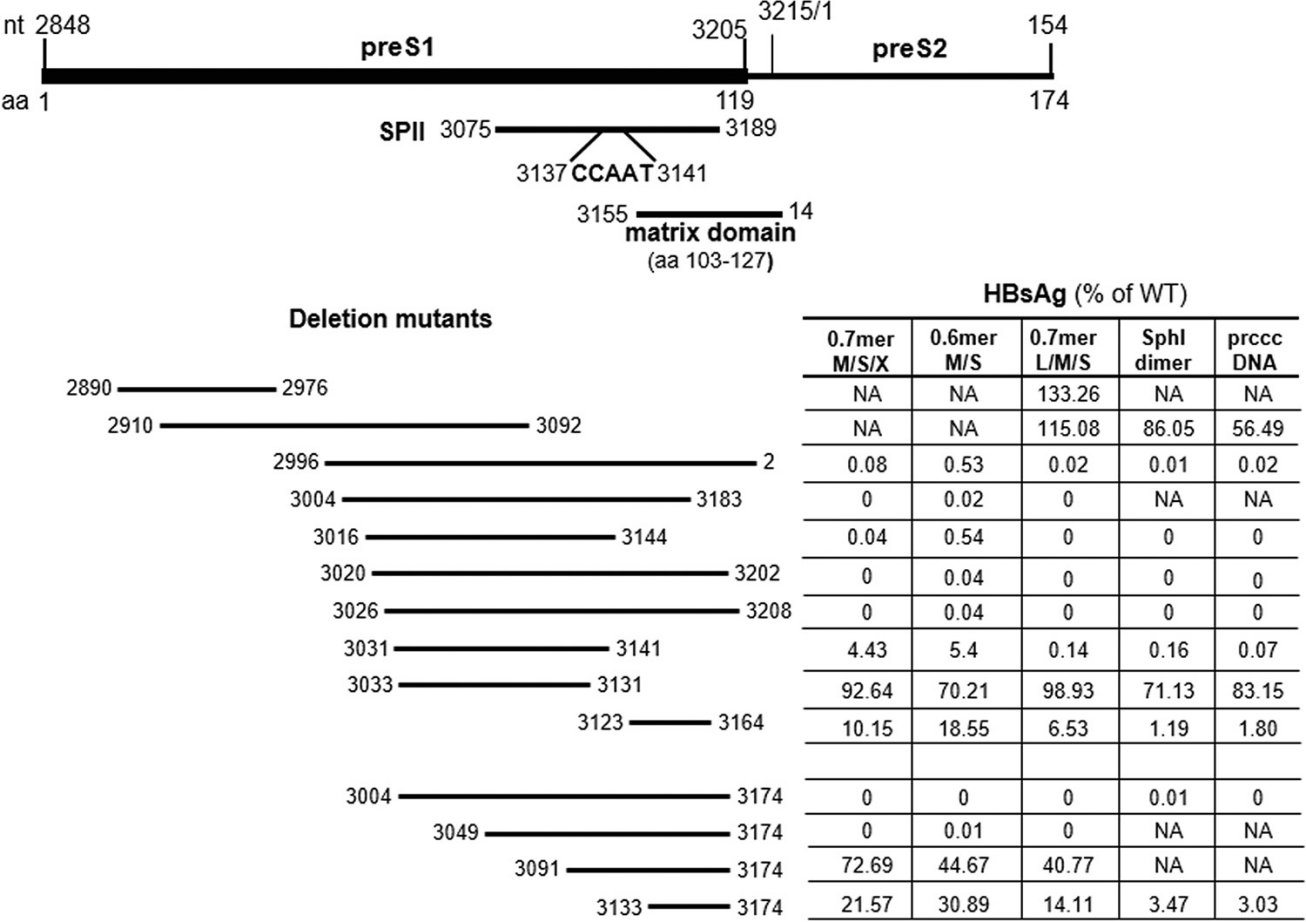
The 14 preS1 deletion mutants characterized in the present study and their efficiencies of HBsAg production from five different types of constructs. The preS1 region (nucleotides 2848 to 3204 for genotype C) encodes amino acids 1 to 119 of L protein. The preS2 region (nucleotides 3205 to 3215 and 1 to 154) encodes amino acids 120 to 174 of the L protein and 1 to 55 of the M protein. The preS region also harbors the SPII promoter driving transcription of the 2.1-kb RNA, with the CCAAT sequence being a critical regulatory element. At the amino acid level, the matrix domain of L protein (preS amino acids 103 to 127) is required for virion morphogenesis. The 14 preS deletion mutants (with the top 10 being naturally occurring) were introduced into clone geno27.2 of genotype C. The impact of these deletions on HBsAg production from different types of constructs is shown to the right (averaged from at least three transfection experiments). NA, not available; no data were available because the mutant was not introduced into that type of construct.

A panel of 10 in-frame preS deletions found in clinical samples (37–39, 41–45, 51, 57) was analyzed (Table 1 and Fig. 2); most were within the preS1 region, but two extended to the 5′ end of the preS2 region (del2996–2 and del3026–3208). In addition, we constructed four artificial deletion mutants with variable 5′ ends but fixed 3′ ends at nucleotide 3174 (Fig. 2, bottom). Considering the high prevalence of preS deletions in genotype C (37, 38, 42), these deletions were introduced into geno27.2, a WT genotype C clone (65, 66). Since the entire envelope gene is overlapped by the P gene (Fig. 1A), deletions at the nucleotide level were accompanied by internal truncation of both L and P proteins (Table 1).

**TABLE 1:** Deletions at the nucleotide and amino acid levels in L and P proteins

Deletion positions	No. of deleted nucleotides	preS domain of L protein		Spacer of P protein		Reference(s)
		Deletion positions	Substitution	Deletion positions	Substitution	
Naturally occurring						
2890–2976	87	15–43		196–224	E195G	39
2910–3092	183	22–82		202–262		38
2996–2	222	50–123	S124A	231–304		38
3004–3183	180	53–112		234–283	A233Q	45
3016–3144	129	57–99		238–280		41, 57
3020–3202	183	58–118		239–299		37, 38, 42–44, 51
3026–3208	183	60–120	Q121E	241–301	S240R	38
3031–3141	111	62–98		243–279	I242T	37
3033–3131	99	63–95		243–275		38
3123–3164	42	93–106		273–286		39
Artificial						
3004–3174	171	53–109		234–290		
3049–3174	126	68–109		249–290	H248P	
3091–3174	84	82–109		263–290		
3133–3174	42	96–109		277–290	L276P	

### Findings from 0.6-mer M/S constructs and 0.7-mer M/S/X constructs suggested that many 3′ preS1 deletions abolished S protein expression at the transcript level.

**FIG 3:**
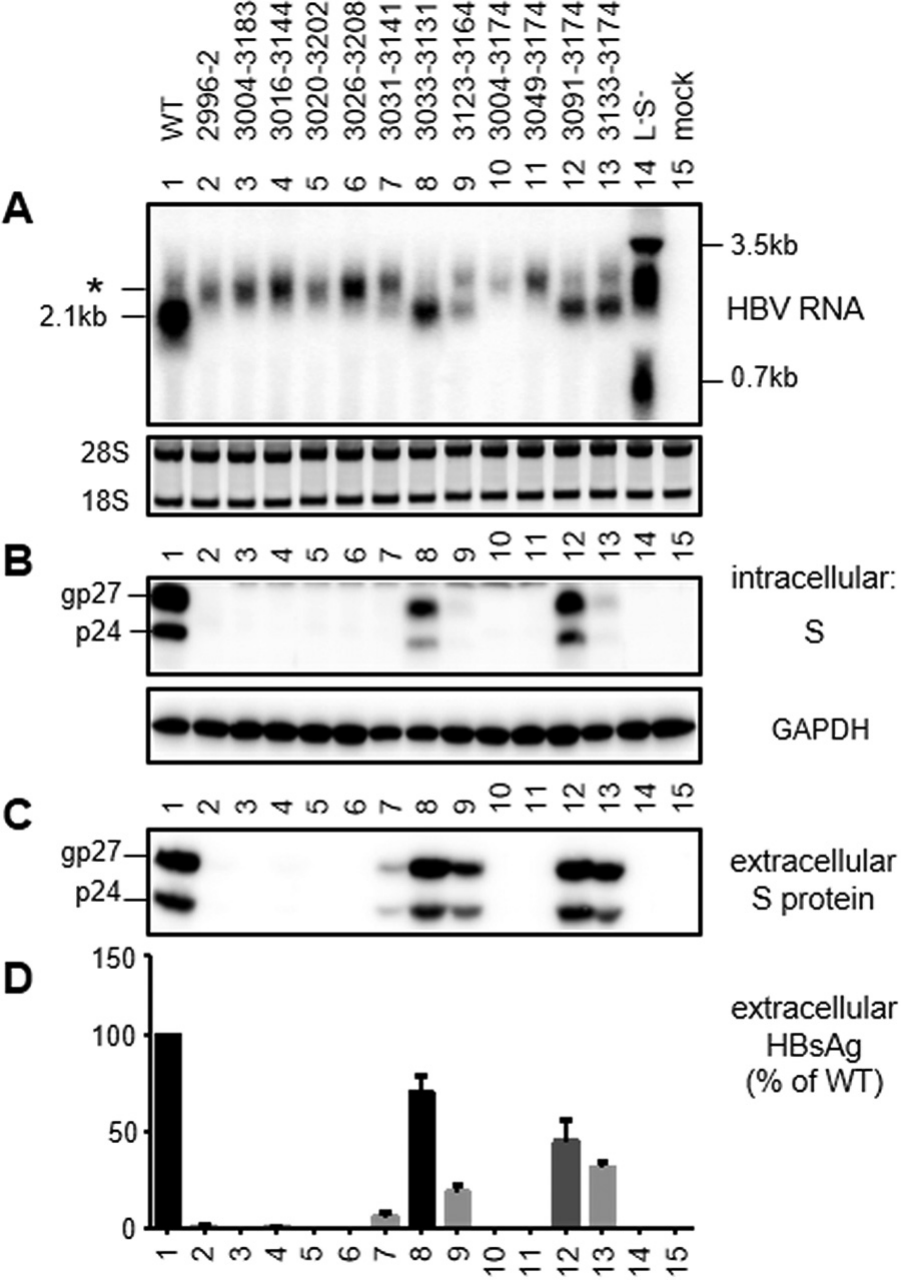
RNA transcription, S protein expression, and HBsAg secretion from 0.6-mer M/S constructs. The WT 0.6-mer construct and its 12 deletion mutants were transiently transfected in Huh7 cells. The L-minus S-minus 1.1-mer construct of geno17.3 served as a negative control for HBsAg production and a size marker for different forms of HBV RNAs. Cells were harvested at day 3 posttransfection for RNA analysis (A) or day 4 posttransfection for protein analysis (B to D). (A) Northern blot analysis of HBV RNA. A probe generated from the full-length HBV genome was used. For the 0.6-mer construct, the 2.1-kb band indicates RNA of the correct size, while the asterisk indicates a band of slower mobility associated with deletion mutants unable to produce HBsAg. Ethidium bromide staining of the 28S and 18S rRNAs served as a loading control. (B) Western blot analysis of intracellular S protein, with GAPDH serving as a loading control. gp27 and p24 are glycosylated and nonglycosylated forms of S protein, respectively. (C) Western blot analysis of secreted S protein following PEG precipitation of SVPs from culture supernatant. (D) ELISA of secreted HBsAg. Data were averaged from three transfection experiments, with the value for the WT construct set as 100%.

With the exception of del2890–2976, all of the deletions covered partially or completely the SPII promoter (nucleotides 3075 to 3189) (Fig. 2) (61, 62). Considering that reduced S protein expression increases the L/S protein ratio to further diminish HBsAg secretion (14–17), we initially employed a 0.6-mer M/S construct to avoid complication from L protein. The construct contained nucleotides 2954 to 3215 and 1 to 835 of genotype C, covering both the SPII promoter and coding sequences for both M and S proteins, followed by a posttranscriptional regulatory element (nucleotides 970 to 1770) (67, 68) to facilitate cytoplasmic export of the transcript and a polyadenylation signal from SV40 (Fig. 1C). It could not express the authentic HBx protein of 154 aa due to a 3′ deletion in the X gene (nucleotides 1374 to 1838). Instead, the N-terminal 132 aa of HBx protein would be joined by 70 aa of irrelevant sequence. From the 0.6-mer M/S construct, the amount of HBsAg secreted should be directly proportional to the amounts of S and M proteins expressed, which in turn should be controlled at the transcript level. Two deletion mutants (del2890–2976 and del2910–3092) were not analyzed as 0.6-mer M/S constructs, because the 5′ ends of their deletions lay upstream of the HBV insert. In transiently transfected Huh7 cells, mutants del3033–3131, del3091–3174, del3133–3174, del3123–3164, and del3031–3141 showed progressive reductions in the amounts of HBsAg released, according to enzyme-linked immunosorbent assays (ELISAs) (Fig. 2 and 3D). The other seven mutants produced <1% of the WT level of HBsAg or were HBsAg negative even when undiluted culture supernatant was used (Fig. 2). In agreement with ELISA data, Northern blot analysis revealed RNA bands of the correct size to be WT more than del3033–3131 more than del3091–3174 and del3133–3174 more than del3123–3164 (Fig. 3A). Surprisingly, deletion mutants that were negative for HBsAg production generated an RNA band of higher molecular weight (denoted by an asterisk in Fig. 3A).

**FIG 4:**
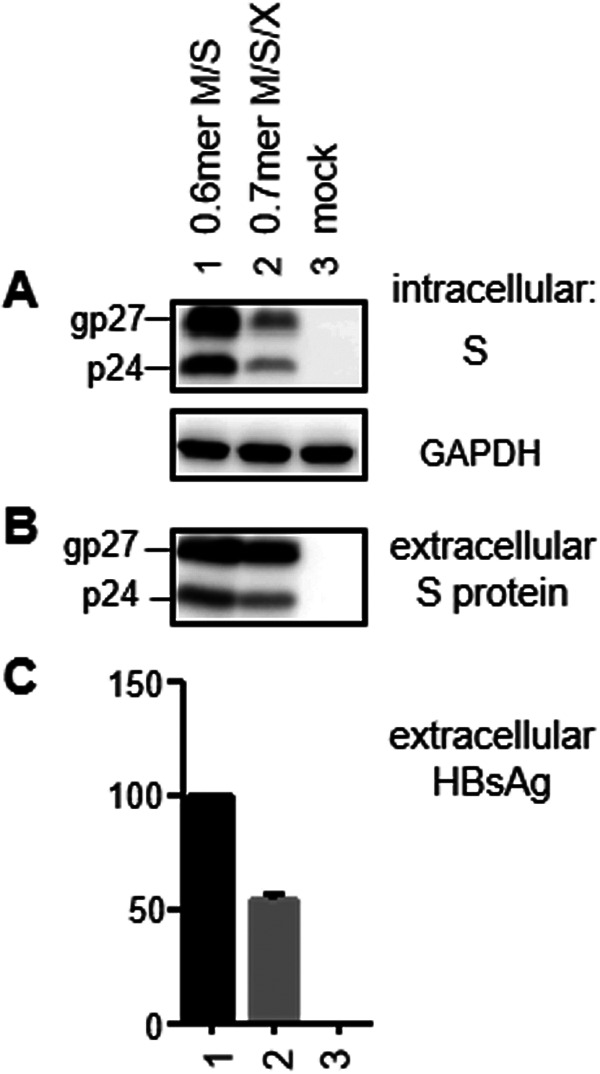
Comparison of S protein expression and HBsAg secretion between the 0.6-mer M/S construct and the 0.7-mer M/S/X construct. Huh7 cells were transfected with the WT 0.6-mer M/S construct or the WT 0.7-mer M/S/X construct. Both cells and culture supernatant were harvested 3 days later. (A) Western blot of intracellular S protein, with GAPDH serving as a loading control. (B) Western blot of secreted S protein following PEG precipitation of SVPs. (C) ELISA of secreted HBsAg (averaged from three transfection experiments, with the value for the 0.6-mer M/S construct set as 100%).

**FIG 5:**
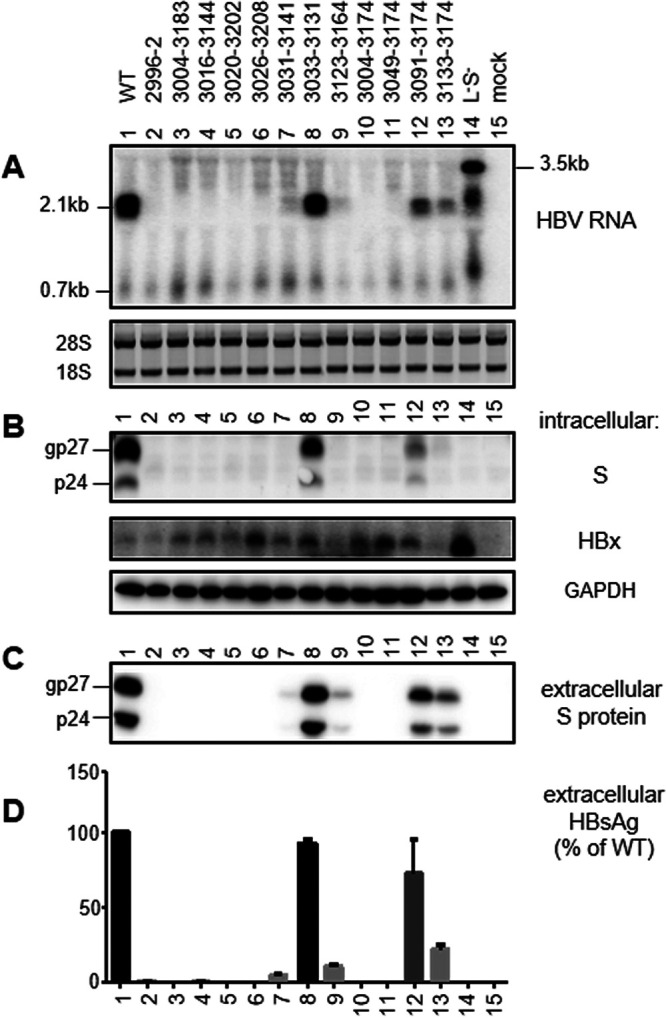
RNA transcription, S and HBx protein expression, and HBsAg secretion from 0.7-mer M/S/X constructs. Huh7 cells were transiently transfected with the WT construct and its 12 deletion mutants. The L-minus S-minus 1.1-mer construct of geno17.3 served as a control. Cells were harvested 3 days (A) or 4 days (B to D) later. (A) Northern blot of HBV RNAs using a 0.7-kb HBV DNA probe. The 28S and 18S rRNAs served as loading controls. (B) Western blot of intracellular S and HBx proteins, with GAPDH serving as a loading control. (C) Western blot of S protein concentrated from the culture supernatant by PEG precipitation. (D) HBsAg in the culture supernatant assessed by ELISA. Data were averaged from three transfection experiments, with the value for the WT construct set as 100%.

To avoid the production of such an RNA band and to enable expression of authentic HBx protein, we made a 0.7-mer M/S/X construct by inserting into the pBluescript SK(−) vector a single 2.2-kb HBV DNA fragment (nucleotides 2954 to 3215 and 1 to 1960 of geno27.2) covering the entire HBx coding sequence and the endogenous poly(A) signal downstream (Fig. 1C). For the WT sequence, such a 0.7-mer M/S/X construct produced less S protein than the corresponding 0.6-mer M/S construct and secreted less HBsAg (Fig. 4). However, similar to the 0.6-mer M/S construct, the 0.7-mer M/S/X construct secreted HBsAg in the order of WT more than del3033–3131 more than del3091–3174 more than del3133–3174 more than del3123–3164 more than del3031–3141 (Fig. 2 and 5D). Northern blotting revealed the amount of the 2.1-kb RNA to be WT more than del3033–3131 more than del3091–3174 more than del3133–3174 (Fig. 5A). Importantly, the seven deletion mutants that were deficient in HBsAg production lost the 2.1-kb RNA without producing any band of higher molecular weight.

### Identification of nucleotides 3085 to 3090 in the 5′ SPII promoter as a positive regulatory element for HBsAg production.

**FIG 6:**
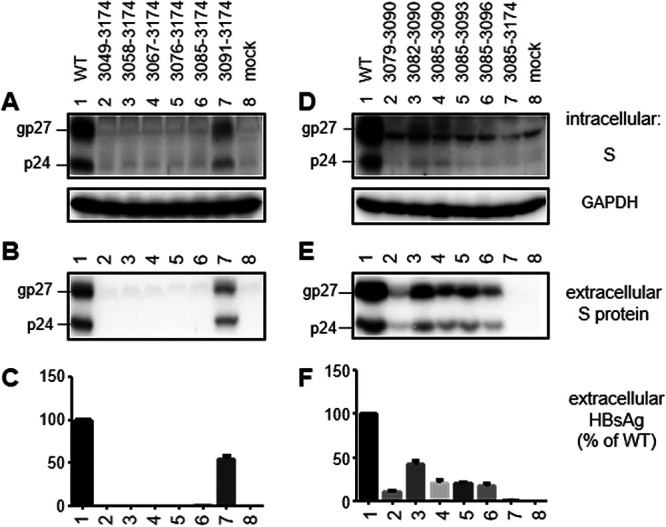
Deletion mutants of 0.7-mer M/S/X constructs to map a sequence in the SPII promoter critical for efficient S protein expression. (A to C) Four new deletion mutants to map the 5′ boundary of a positive regulator of S protein expression. (D to F) Validation of the contribution of nucleotides 3085 to 3090 to efficient S protein expression by precise removal of that sequence or additional sequences upstream or downstream. Huh7 cells were transiently transfected with WT construct or various deletion mutants. Cells and culture supernatant were harvested 4 days later. (A and D) Western blots of intracellular S protein, with GAPDH serving as a loading control. (B and E) Western blots of S protein concentrated from the culture supernatant. (C and F) HBsAg in the culture supernatant assessed by ELISA. Data were averaged from three independent transfection experiments, with the value for the WT construct set as 100%.

Among the four artificial mutants with fixed 3′ ends of deletion but variable 5′ ends, del3091–3174 continued to produce high levels of HBsAg as either a 0.6-mer M/S construct or a 0.7-mer M/S/X construct, whereas del3049–3174 became HBsAg negative (Fig. 2). We employed the 0.7-mer M/S/X construct to move the 5′ end of deletion to position 3058, 3067, 3076, or 3085. All of the four new deletion mutants were negative for S protein and HBsAg in culture supernatant (Fig. 6B and C), and precise removal of nucleotides 3085 to 3090 reduced HBsAg production by 80% (Fig. 6F). Extending the 3′ end of the deletion to position 3093 or 3096 had limited impact on HBsAg levels. Extending the 5′ end of deletion by 6 nt (del3079–3090) further reduced HBsAg titers but extending it by just 3 nt (del3082–3090) rather increased titers (Fig. 6F), suggesting nucleotides 3079 to 3081 as a positive regulator of HBsAg production. According to a previous study based on genotype D, nucleotides 3079 to 3081 and nucleotides 3085 to 3090 for genotype C were located inside positive transcriptional regulatory regions B and C, respectively, of the SPII promoter (62).

### Findings from the 0.7-mer L/M/S constructs, SphI dimers, and prcccDNA constructs supported the CCAAT element inside the SPII promoter as a positive regulator of 2.1-kb RNA but a negative regulator of 2.4-kb RNA.

**FIG 7:**
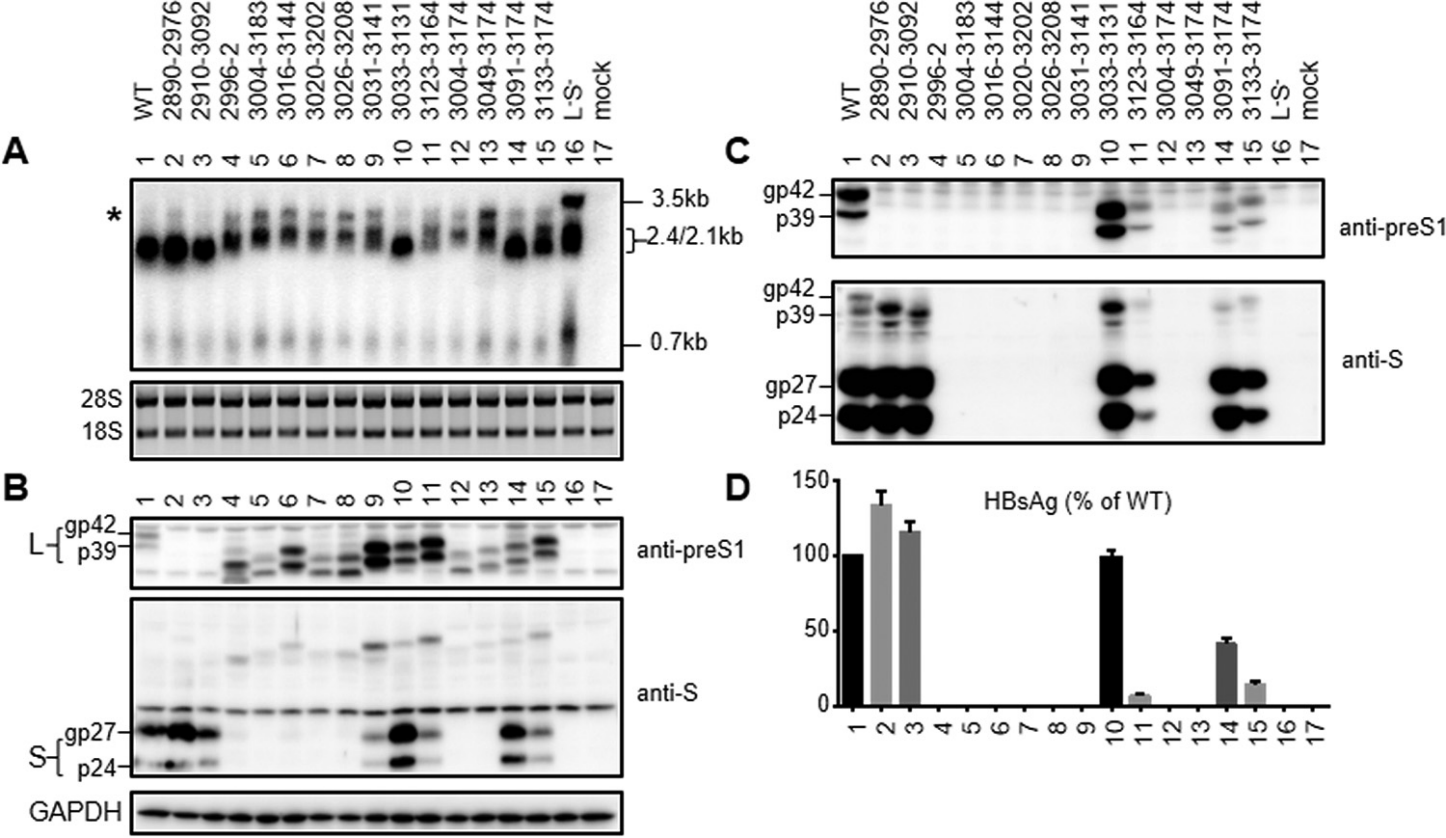
RNA transcription, L and S protein expression, and HBsAg secretion from 0.7-mer L/M/S constructs. Huh7 cells were transiently transfected with the WT construct, 14 deletion mutants, and a L-minus S-minus 1.1-mer construct. (A) Northern blot analysis of HBV RNAs at day 3 posttransfection using the 0.7-kb HBV DNA probe. The asterisk indicates a band of slow mobility that is dominant from deletion mutants unable to produce HBsAg. The 28S and 18S rRNAs served as loading controls. (B to D) HBV envelope proteins and HBsAg detected at day 4 posttransfection. (B) Intracellular L and S proteins detected by polyclonal rabbit anti-preS1 antibody (upper) and anti-S antibody (middle), respectively. GAPDH served as a loading control (lower). gp42 and p39 are glycosylated and nonglycosylated forms of L protein (from the WT construct), respectively. (C) Extracellular L and S proteins following PEG precipitation of SVPs, detected by anti-preS1 and anti-S antibodies, respectively. (D) ELISA of extracellular HBsAg. Data were averaged from four transfection experiments, with the value for the WT construct set as 100%.

Next, we generated 0.7-mer L/M/S constructs for WT virus and the 14 deletion mutants to establish the impact of preS1 deletions on HBsAg titers in the context of L protein expression. The constructs had an extended 5′ end of the HBV insert, relative to the 0.6-mer M/S constructs, to enable L protein expression under the SPI promoter (Fig. 1C). Del2910–3092 and especially del2890–2976 secreted more HBsAg than the WT construct (Fig. 7D). Both intracellular 2.1-kb RNA and S protein were increased in del2890–2976 but reduced in del2910–3092 (Fig. 7A and B); no L protein could be detected in cell lysate or culture supernatant from these two mutants (Fig. 7B and C), which had deletions of preS1 amino acids 15 to 43 and amino acids 22 to 82, respectively (Table 1). In this regard, the rabbit polyclonal antibody used for Western blotting was raised against preS1 amino acids 12 to 46 of genotypes B and C (69), suggesting a lost antigenic epitope. Western blotting with anti-S antibody revealed that more L protein was secreted from these two mutants than from the WT construct (Fig. 7C). Thus, del2890–2976 increased HBsAg production at the transcript level, whereas del2910–3092 probably promoted HBsAg secretion through a weakened inhibitory effect of the truncated L protein.

**FIG 8:**
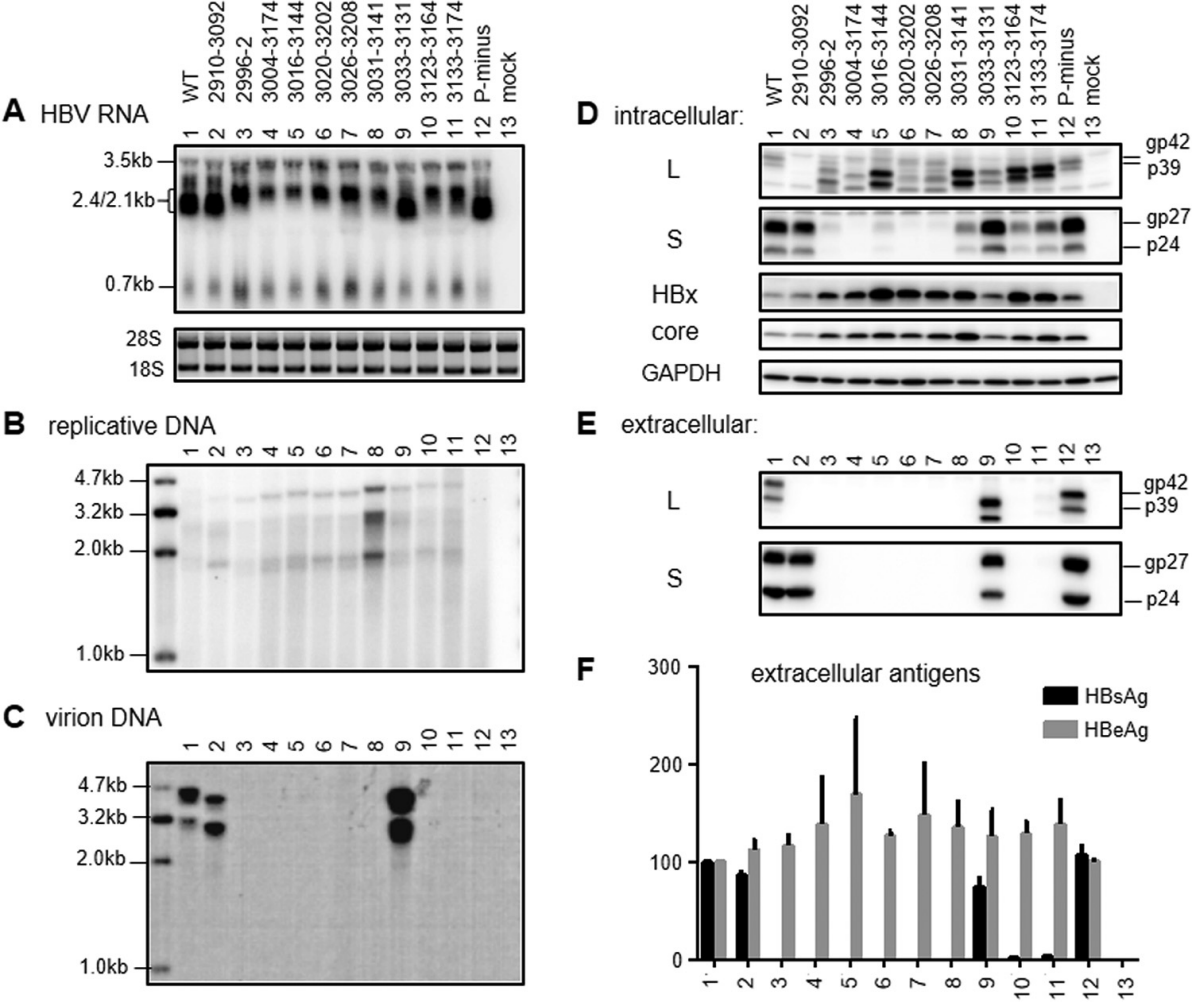
RNA transcription, protein expression, genome replication, and virion and SVP secretion from SphI dimer constructs. Huh7 cells were transfected with the SphI dimers of WT virus, 10 preS1 deletion mutants, and a P-minus mutant to serve as a negative control for genome replication. Cells were harvested 3 days later for RNA analysis (A) or 5 days later for DNA and protein analysis (B to F). (A) Northern blot analysis of intracellular HBV RNAs. (B) Southern blot analysis of intracellular replicative DNA. (C) Southern blot analysis of virion DNA following immunoprecipitation of virions from culture supernatant with anti-S antibody. The Northern and Southern blots were hybridized with a 0.7-kb and full-length HBV DNA probe, respectively. (D) Western blot analysis of intracellular L, S, HBx, and core proteins, using GAPDH as a loading control. (E) Western blot analysis of secreted L and S proteins following PEG precipitation of virions and SVPs. (F) ELISA of secreted HBsAg and HBeAg. Data were averaged from four transfection experiments, with the value for the WT construct set as 100%.

**FIG 9:**
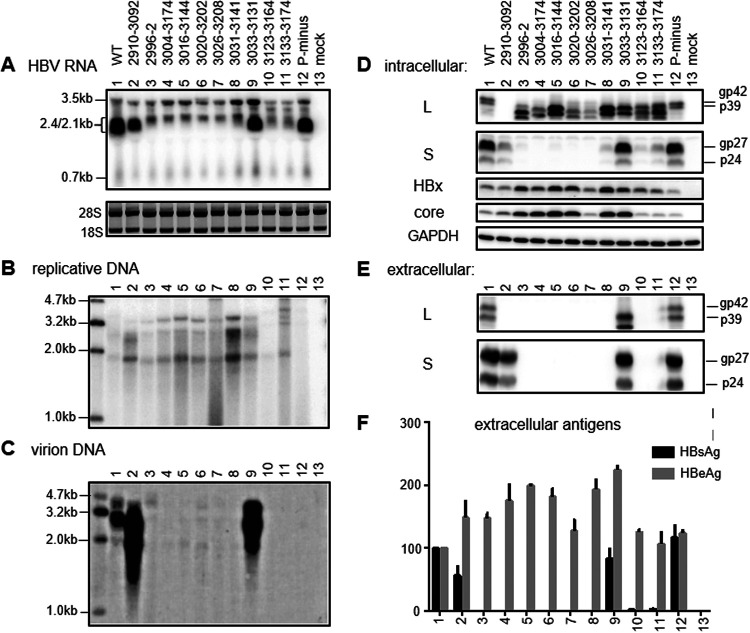
RNA transcription, protein expression, genome replication, and virion and SVP secretion from prcccDNA constructs. Huh7 cells were transfected with the prcccDNA constructs and harvested 3 days later for RNA analysis (A) or 5 days later for DNA and protein analysis (B to F). (A) Northern blot analysis of intracellular HBV RNAs using the 0.7-kb probe. (B and C) Southern blot analysis of intracellular replicative DNA (B) and extracellular virion DNA (C) using the full-length probe. (D) Western blot analysis of intracellular L, S, HBx, and core proteins using GAPDH as a loading control. (E) Western blot analysis of secreted L and S proteins following PEG precipitation of virions and SVPs. (F) ELISA of secreted HBsAg and HBeAg. Data were averaged from three transfection experiments, with the value for the WT construct set as 100%.

Of the 12 deletion mutants already characterized as 0.6-mer M/S constructs and 0.7-mer M/S/X constructs, all seven mutants deficient in HBsAg production (<1% of WT level) remained HBsAg negative as 0.7-mer L/M/S constructs (Fig. 2 and 7F). Five of these (del2996–2, del3004–3174, del3016–3144, del3020–3202, and del3026–3208) were further analyzed as SphI dimers (with tandem copies of the HBV genome cloned to a vector via the unique SphI site in the HBV genome) and precursor to recombinant cccDNA (prcccDNA) constructs (to mimic cccDNA), and all remained HBsAg negative (Fig. 8F and 9F). Of deletion mutants with reduced HBsAg production as 0.6-mer M/S constructs, del3123–3164 and del3133–3174 showed further reduction as 0.7-mer L/M/S constructs and even greater reduction as SphI dimers and prcccDNA constructs (Fig. 2). HBsAg secretion from del3031–3141, already low for the 0.6-mer M/S construct (5.4% of WT level), became nearly negative in the three new types of constructs. All three deletion mutants lost the CCAAT element (nucleotides 3137 to 3141) (Fig. 2), which was previously found to markedly enhance transcription of the 2.1-kb RNA while suppressing transcription of the 2.4-kb RNA (70–72). This element was retained in del3033–3131, which continued to secrete high levels of HBsAg even as full-length constructs (Fig. 2). Northern blot analysis revealed upshift of the 2.4-kb/2.1-kb RNA band for deletion mutants with lost HBsAg production, suggesting increased transcription of the 2.4-kb RNA (Fig. 7A, 8A, and 9A). Similar to 0.6-mer M/S constructs, the 0.7-mer L/M/S constructs produced another band of even slower migration when HBsAg production was lost (Fig. 7A, asterisk).

### Complete loss of the 2.1-kb RNA/S protein was often not accompanied by marked increases of intracellular L protein despite upshift of the 2.4-kb/2.1-kb RNA band and stalled L protein secretion.

**FIG 10:**
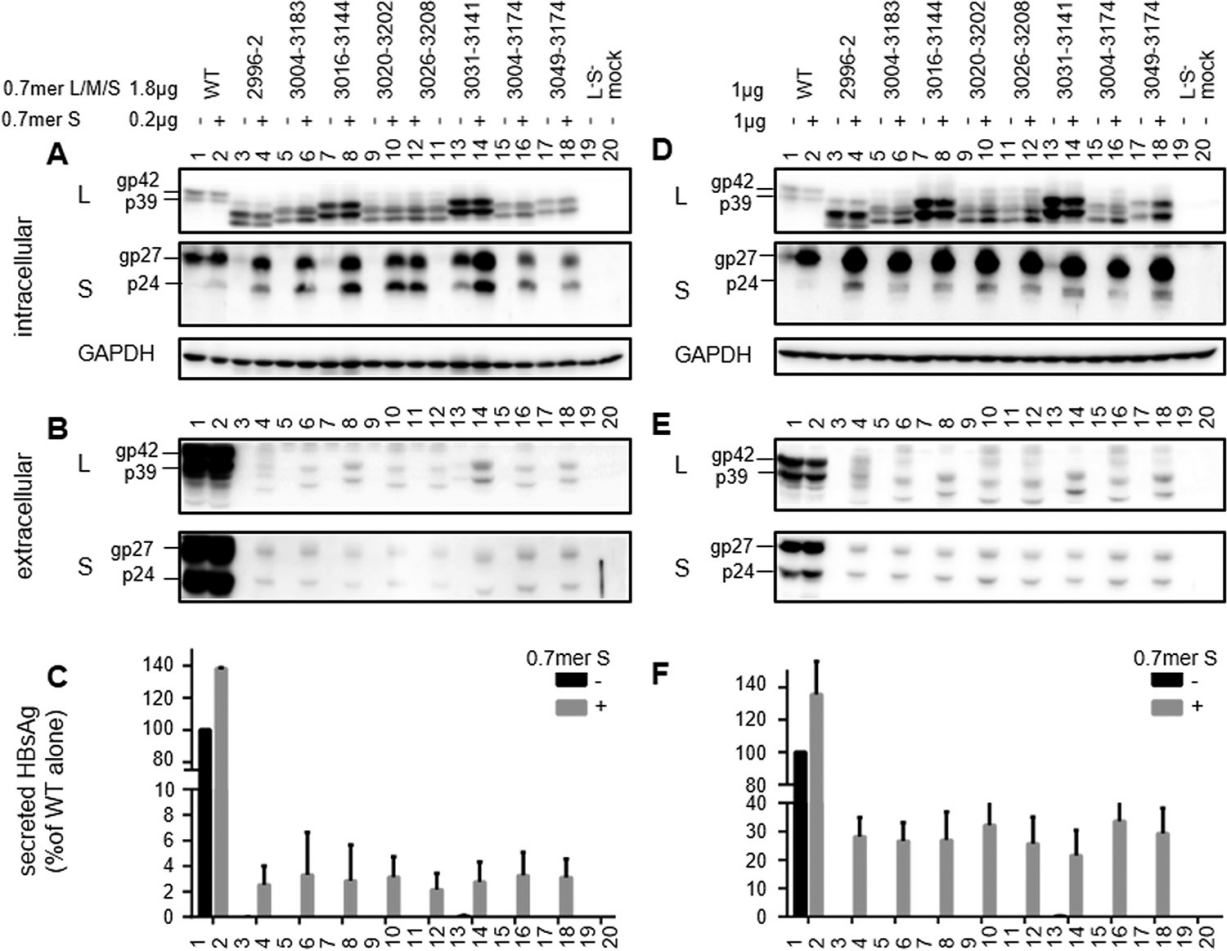
Full-length S protein provided in *trans* rescued L protein secretion from 0.7-mer L/M/S constructs with impaired HBsAg production. The parental construct (WT) and its eight preS deletion mutants with impaired secretion as 0.7-mer L/M/S constructs were cotransfected with the S protein expression construct N65 at a ratio of 1.8 μg to 0.2 μg (A to C) or 1 μg to 1 μg (D to F), using pBluescript SK(−) as a negative control. The transfected Huh7 cells were harvested 4 days later. (A and D) Western blots of intracellular L and S proteins, with GAPDH serving as a loading control. (B and E) Western blots of extracellular L and S proteins following PEG precipitation of SVPs. (C and F) ELISA of secreted HBsAg. Data were averaged from three transfection experiments, with the value for the WT construct without cotransfection with N65 set as 100%.

For the seven deletion mutants with lost HBsAg secretion even as 0.6-mer M/S constructs (del2996–2, del3004–3183, del3016–3144, del3020–3202, del3026–3208, del3004–3174, and del3049–3174), the corresponding 0.7-mer L/M/S constructs had clear upshifts of the 2.4-kb/2.1-kb RNA band (Fig. 7A). However, they showed only modest increases of intracellular L protein despite its secretion block due to lack of S protein coexpression (Fig. 7B and 10B). The highest levels of L protein were rather achieved by del3031–3141 and del3123–3164, both retaining low levels of intracellular S protein (Fig. 7D). For SphI dimers and prcccDNA constructs, high intracellular levels of L protein were also achieved by del3133–3174 (with much reduced HBsAg production, compared with the 0.7-mer L/M/S construct) and del3016–3144 (HBsAg negative) (Fig. 8D and 9D). To test the ability of S protein to sustain high intracellular levels of L protein, the 0.7-mer L/M/S constructs of WT virus and the eight deletion mutants with lost HBsAg production were cotransfected with a 0.7-mer S protein expression construct at a 9:1 or 1:1 ratio. Coexpressed S protein was detectable in cell lysate and secreted to culture supernatant (Fig. 10A and D and Fig. 10B and E, respectively), leading to positive HBsAg signals (Fig. 10C and F). The mutant L proteins with internal deletions were also secreted, which was more efficient at a 1:1 ratio than at a 9:1 ratio of cotransfection (Fig. 10B and E). Intracellular levels of L protein increased for some deletion mutants, especially at a 1:1 ratio (Fig. 10A and D). No increase or decrease was observed for deletion mutants with high intracellular levels of L protein to begin with (del3016–3144 and del3031–3141), and their secretion in the presence of exogenous S protein was not greater than that of other mutants (Fig. 10B and E).

### Lost HBsAg production was often accompanied by increased HBx protein expression from full-length constructs.

**FIG 11:**
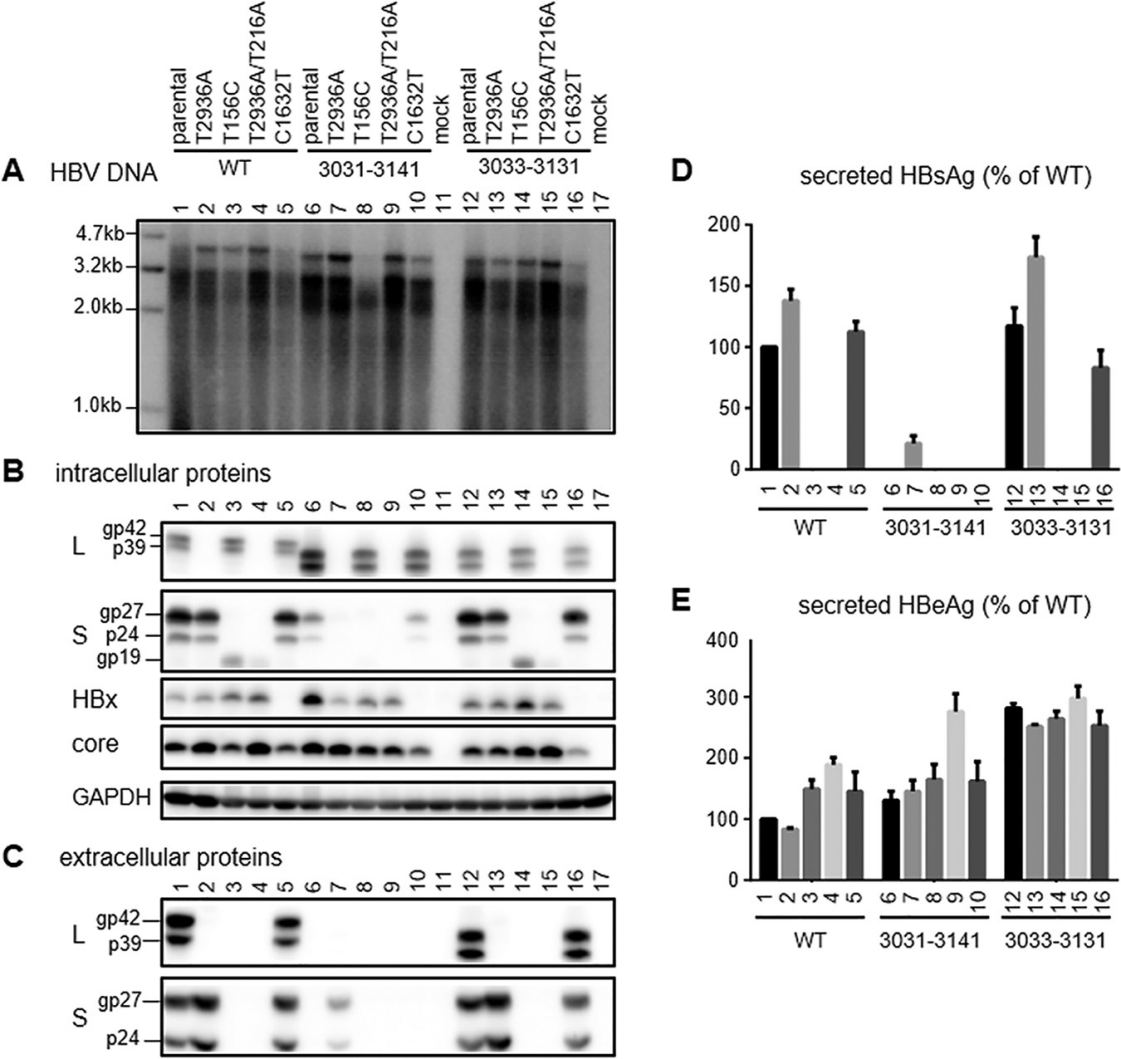
Genome replication, protein expression, and HBsAg and HBeAg secretion following transfection with site-directed mutants of SphI dimer constructs in Huh7 cells. Cells were transfected with the SphI dimer of the parental WT construct, the del3031–3141 or del3033–3131 deletion mutant, or their L-minus (T2936A), S-minus (T156C), L/S-minus (T2936A/T216A), or X-minus (C1632T) mutant. Cells and culture supernatant were harvested 5 days later. (A) Southern blot analysis of intracellular replicative DNA. (B) Western blot analysis of intracellular L, S, HBx, and core proteins, using GAPDH as a loading control. gp19 is a truncated S protein through translation initiation from codon 75 (M75). (C) Western blot analysis of secreted L and S proteins following PEG precipitation. (D) ELISA of secreted HBsAg. (E) ELISA of secreted HBeAg. Data were averaged from three transfection experiments, with the value for the WT construct set as 100%.

**FIG 12:**
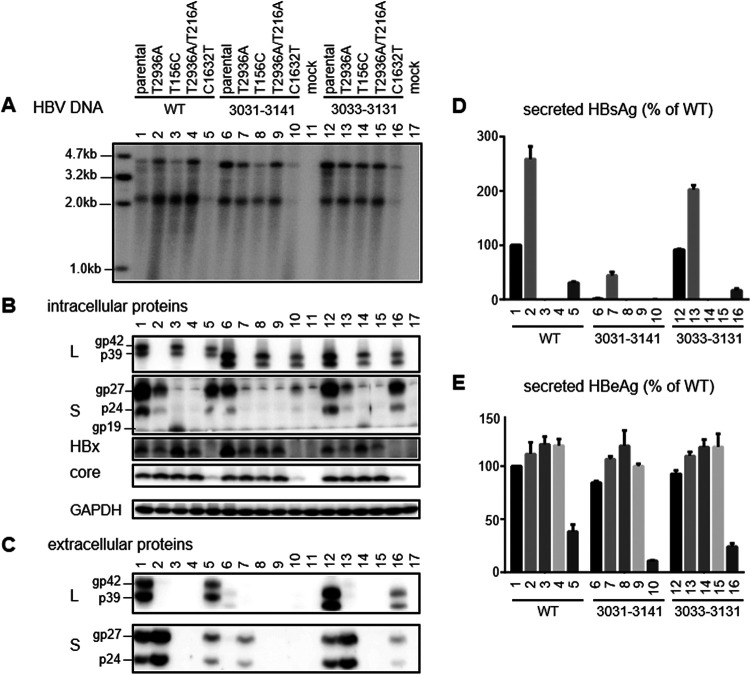
Genome replication, protein expression, and HBsAg and HBeAg secretion following transfection with site-directed mutants of SphI dimer constructs in HepG2 cells. Cells were transfected with the SphI dimer of the parental WT construct, del3031–3141 or del3033–3131 deletion mutant, or their L-minus (T2936A), S-minus (T156C), L/S-minus (T2936A/T216A), or X-minus (C1632T) mutant. Cells and culture supernatant were harvested 5 days later. (A) Southern blot analysis of intracellular replicative DNA. (B) Western blot analysis of intracellular L, S, HBx, and core proteins, using GAPDH as a loading control. (C) Western blot analysis of secreted L and S proteins following PEG precipitation. (D) ELISA of secreted HBsAg. (E) ELISA of secreted HBeAg. Data were averaged from three transfection experiments, with the value for the WT construct set as 100%.

Transcriptional interference among coterminal HBV RNAs would predict increased 0.7-kb RNA levels when the 2.1-kb RNA is no longer transcribed (6–8, 73). The 0.7-kb RNA could be detected in Northern blots with a ^32^P-labeled DNA probe based on the corresponding DNA fragment rather than the full-length genome (Fig. 5A), and some deletion mutants with lost HBsAg production as 0.7-mer M/S/X constructs (such as del3004–3183 and del3031–3141) showed increased intensity of the 0.7-kb RNA. Western blot analysis revealed increased HBx protein for mutants like del3026–3208 and del3049–3174 (Fig. 5B), although the mRNA and protein levels did not show a clear correlation. In this regard, the monoclonal antibody (MAb) used for Western blotting, 20F3, targeted amino acids 102 to 119 in HBx protein. This protein band was abolished by a C1632T nonsense mutation (Q87* at the protein level) introduced into the SphI dimer of WT HBV and two preS1 deletion mutants (Fig. 11B and 12B), thus confirming its specificity. For SphI dimers, all of the deletion mutants with undetectable HBsAg in culture supernatant showed increased HBx protein (Fig. 8D), and Northern blotting revealed increased 0.7-kb RNA (Fig. 8A). Increases of HBx protein were also observed for most such deletion mutants as prcccDNA constructs (Fig. 9D).

### Full-length constructs of HBsAg-negative deletion mutants were deficient in virion secretion but often produced more 3.5-kb RNA, core protein, and replicative DNA.

Northern blotting revealed increased 3.5-kb RNAs from SphI dimers of all of the preS deletion mutants with no or little HBsAg secretion but not del2910–3092, with sustained HBsAg production (Fig. 8A). The 3.5-kb RNA is subdivided into pcRNA for HBeAg expression and pgRNA driving core/P protein translation as well as genome replication. Increased HBeAg secretion from the eight mutants with impaired HBsAg production was variable and modest for SphI dimers but more pronounced for prcccDNA constructs (Fig. 8F and 9F). For SphI dimers, the eight mutants with impaired HBsAg production but not mutants with sustained HBsAg production (such as del2910–3092 and del3033–3131) had increased intracellular core protein (Fig. 8D); for prcccDNA, the reverse correlation between HBsAg and core protein was less strict (Fig. 9D). Most mutants with impaired HBsAg production had higher intracellular levels of replicative DNA than the WT construct, with the highest level achieved by del3031–3141 for both SphI dimer and prcccDNA (Fig. 8B and 9B). This mutant was deficient in virion production (Fig. 8C and 9C). Other deletion mutants with complete lack of HBsAg production also released no or few virions. Both del2910–3092 and del3033–3131, with sustained HBsAg production, showed efficient virion secretion (Fig. 8C and 9C).

### Examining the contributions of envelope and HBx proteins to the high intracellular replicative DNA of the del3031–3141 mutant shows a greater impact of HBx protein on HBV genome replication and protein expression in HepG2 cells than in Huh7 cells.

Del3031–3141 was similar to many other deletion mutants in impaired transcription of 2.1-kb RNA, which might increase the 3.5-kb pgRNA to augment genome replication. Alternatively, increased HBx expression could stimulate transcription of pgRNA to enhance genome replication. Impaired S protein expression would also prevent virion secretion, which could retain the enveloped or nonenveloped core particles to increase intracellular replicative DNA and core protein. Del3031–3141 was unusual in having a higher intracellular level of L protein and unique in having residual S protein expression, which might stabilize core particles better than L protein alone. Therefore, we generated a HBx-minus mutant of del3031–3141 by using the Q87* nonsense mutation (C1632T at the nucleotide level), an L-minus mutant by using the L30* mutation in the preS1 domain (T2936A), an S-minus mutant by converting the ATG codon to ACG (T156C), and an L/M/S-minus mutant by combining the L30* mutation in the preS1 domain with the L21* nonsense mutation (T216A) in the S domain. The same set of mutations was introduced into the WT construct as well as del3033–3131, which had a deletion similar to that of del3031–3141 but sustained HBsAg production and virion secretion. In transfected Huh7 cells, preventing expression of full-length S protein reduced replicative DNA more dramatically for del3031–3141 and least for del3033–3131 (Fig. 11A). Preventing expression of all three envelope proteins increased genome replication for del3033–3131 and especially the WT construct but had limited impact on del3031–3141. Preventing HBx protein expression reduced genome replication for all three constructs, although del3031–3141 continued to display the highest level of replicative DNA. At the protein level, preventing S protein expression reduced intracellular L protein for del3031–3141 but increased HBx and core proteins for del3033–3131 (Fig. 11B). As expected, preventing L protein expression increased extracellular S protein and HBsAg at the expense of intracellular S protein, leading to HBsAg secretion by del3031–3141 similar to that of its 0.6-mer M/S construct and 0.7-mer M/S/X construct (Fig. 11B to D). Preventing expression of all three envelope proteins increased intracellular core protein for both the WT construct and the del3033–3131 mutant, while lost HBx protein expression reduced intracellular levels of S protein and in particular core protein (Fig. 11B).

The results presented above demonstrated a role of HBx protein in augmenting core protein expression and HBV genome replication in Huh7 cells. When the same set of mutants were transfected into HepG2 cells, the HBx-minus mutants displayed even greater reduction in genome replication and core and S protein expression, compared with the three parental constructs. Moreover, the HBx-minus mutants even showed clear reductions of intracellular and extracellular L protein, secreted HBsAg, and even HBeAg (Fig. 12). In this cell line, the HBx-minus mutation reduced replication of del3031–3141 to a greater extent than the S-minus mutation (Fig. 12A).

## DISCUSSION

We previously found that naturally occurring A1762T/G1764A/C1766T and T1753C/A1762T/G1764A/C1766T CPMs markedly enhanced HBV genome replication while suppressing HBeAg expression in the context of a dimeric genotype A clone (26). The dual effect was attributed to greatly increased transcription of pgRNA at the expense of slightly longer pcRNA (63). Interestingly, intracellular S protein and extracellular HBsAg were moderately reduced by such mutations in transiently transfected Huh7 cells, although the impact on HBx protein expression was not examined. Northern blotting revealed increased 3.5-kb RNA but reduced 2.4-kb/2.1-kb and 0.7-kb RNAs (64). Further evidence for transcriptional interference from the 3.5-kb pgRNA against subgenomic RNAs was obtained by inserting the cDNA equivalent of pgRNA into the pcDNA3.1/Zeo(−) vector downstream of the CMV promoter to generate a 1.1-mer construct, thus permitting pgRNA overproduction. Deleting the CMV promoter from such a construct to prevent pgRNA transcription markedly increased both 2.4-kb/2.1-kb and 0.7-kb RNAs. In this regard, preventing pgRNA transcription from the related duck HBV increased both subgenomic transcripts (one for L protein and the other for S protein) (74, 75), suggesting that transcriptional interference of subgenomic RNAs by pgRNA is a shared feature of this group of hepatotropic DNA viruses.

Transcriptional interference is often unidirectional, with the upstream transcription unit inhibiting transcription from the downstream unit through promoter occlusion (6–8). If this were the case, then transcription of the 2.1-kb HBV RNA should inhibit only 0.7-kb RNA production. During natural infection, however, HBV RNA transcription is driven by the cccDNA, from which the 3′ end of all subgenomic transcripts overlaps the 5′ end of the 3.5-kb RNA (Fig. 1A). Hence, from cccDNA templates, the 2.1-kb RNA is downstream of the 2.4-kb RNA, upstream of the 0.7-kb RNA, but either downstream or upstream of the 3.5-kb RNA, depending on temporal effects. From SphI dimers (with insertion of foreign sequence), the transcription unit for the 2.1-kb RNA lies downstream of the 3.5-kb RNA (Fig. 1B). According to transcriptional interference through promoter occlusion, lost transcription of 2.1-kb RNA from SphI dimers should increase the 0.7-kb RNA but not necessarily the 2.4-kb or 3.5-kb RNA. Therefore, we added prcccDNA constructs to mimic the cccDNA (76).

Transcription of the 2.1-kb RNA is driven by the SPII promoter, which for genotype D has been mapped to a 200-nt fragment in the preS1 region, with the 3′ 115 nt being essential (61, 62); that corresponds to nucleotides 3075 to 3189 in the genotype C genome (Fig. 2). We selected 10 naturally occurring and four artificial preS deletions for functional characterization in the context of a WT genotype C clone. To facilitate data interpretation, three types of subgenomic constructs (0.6-mer M/S, 0.7-mer M/S/X, and 0.7-mer L/M/S) were employed in addition to two types of full-length constructs (SphI dimer and prcccDNA). The 183-nt deletion of nucleotides 3020 to 3202 has been frequently reported (37, 38, 42–44, 51), which could be attributed to direct repeat sequence TCAGG at both nucleotides 3015 to 3019 and nucleotides 3198 to 3202 (42). For the 0.6-mer M/S constructs and 0.7-mer M/S/X constructs, seven deletion mutants (del2996–2, del3004–3183, del3016–3144, del3020–3202, del3026–3208, del3004–3174, and del3049–3174) were completely HBsAg negative or produced less than 1% of the WT level (Fig. 2). Northern blot analysis revealed no 2.1-kb RNA for these mutants as 0.7-mer M/S/X constructs, suggesting lost SPII promoter (Fig. 5A). Consistent with our data, others reported lost HBsAg production from full-length HBV constructs by deleting the equivalent of nucleotides 3020 to 3202 from a genotype A clone and the equivalent of nucleotides 3016 to 3144 from a genotype D clone (57, 58). Lost 2.1-kb RNA from the 0.7-mer M/S/X construct increased 0.7-kb RNA for some of the seven deletion mutants, with some showing increased HBx protein (but not necessarily in agreement with RNA data [Fig. 5A and B]). Five of these seven mutants (del2996–2, del3016–3144, del3020–3202, del3026–3208, and del3004–3174) were further examined as full-length constructs. All showed increased 0.7-kb RNA as SphI dimers, although that was less evident for prcccDNA (Fig. 8A and 9A). More strikingly, HBx protein was elevated for all mutants as SphI dimers and all but one mutant (del3026–3208) as prcccDNA constructs (Fig. 8D and 9D). For mutants capable of HBsAg production, the titers were del3031–3141 less than del3123–3164 less than del3133–3174 less than del3033–3131 for any type of construct (Fig. 2). All of the first three deletion mutants displayed increased HBx protein as SphI dimers. Therefore, lost or much reduced 2.1-kb RNA transcription/HBsAg production from SphI dimers could increase HBx protein expression.

The functional consequences of elevated HBx protein expression in the context of SphI dimers were investigated by a nonsense mutation (Q87*) to prevent translation of full-length protein. Besides del3031–3141 (with high HBx and high replication phenotypes), the same nonsense mutation was introduced into del3033–3131 (with a similar deletion but sustained HBsAg production) and the WT construct. In Huh7 cells, the most striking impact of lost HBx expression was reduced intracellular core protein, followed by reduced replicative DNA (Fig. 11A and B). Intracellular S protein was also somewhat reduced (Fig. 11B). Findings from del3031–3141, del3033–3131, and WT constructs were generally consistent. In HepG2 cells, the phenotypes of core protein and especially replicative DNA became even stronger (Fig. 12A and B). In contrast to Huh7 cells, lost HBx protein expression in HepG2 cells clearly reduced HBeAg production (Fig. 11E and 12E). Moreover, both intracellular and extracellular L and S proteins were reduced, with diminished HBsAg secretion (Fig. 12B to D). Data interpretation will be greatly facilitated by Northern blot analysis and primer extension assays to compare the transcriptional impact of lost HBx protein on the pcRNA, pgRNA, 2.4-kb RNA, and 2.1-kb RNA in HepG2 versus Huh7 cells. The greater impact of lost HBx expression on HBV replication in HepG2 cells than in Huh7 cells is consistent with previous reports (77–79). Discordant findings on HBV biological properties in HepG2 versus Huh7 cells raises the question of which human hepatoma cell line transfected with the full-length HBV genome better mimics *in vivo* HBV infection. To date, most HBV transfection experiments have been based on Huh7 cells, due to much greater transfection efficiency, but stable transfection of HepG2 rather than Huh7 cells with sodium taurocholate cotransporting polypeptide (NTCP) enables efficient infection by cell culture-derived HBV particles (69, 80, 81); that should enable the characterization of the biological properties of HBV genetic variants by both transient transfection and infection experiments in HepG2/NTCP cells.

For SphI dimers, the eight deletion mutants with lost (del2996–2, del3004–3174, del3016–3144, del3020–3202, and del3026–3208) or nearly lost (del3031–3141, del3123–3164, and del3133–3174) HBsAg production exhibited moderate increases in 3.5-kb RNA, while del2910–3092, with sustained HBsAg production, did not (Fig. 8A). More strikingly, all eight deletion mutants displayed an upshift of the 2.4-/2.1-kb RNA band, with very strong intensity of the 2.4-kb band for del2996–2, del3026–3208, and del3020–3202 (Fig. 8A). The upshift was also observed for prcccDNA, although the 2.4-kb band was less strong (Fig. 9A). A similar upshift was observed for these deletion mutants as 0.7-mer L/M/S constructs (Fig. 7A), although both the 2.4-kb and 2.1-kb RNAs differed from authentic HBV RNAs in the 3′ end. A deletion of variable length and location should be present in both the 3.5-kb and 2.4-kb RNAs and could affect the RNA level through altered stability. If increased 3.5-kb and 2.4-kb RNAs from SphI dimers were mainly attributed to increased transcription, then that could be attributed to lost transcriptional interference from 2.1-kb RNA at the step of elongation rather than initiation (through promoter occlusion), because the promoters for 2.4-kb RNA and especially 3.5-kb RNA lie upstream. Alternatively, increased 3.5-kb and 2.4-kb RNAs could be secondary to elevated expression of HBx protein, which could upregulate HBV RNA transcription. That possibility could be verified by comparing levels of the 3.5-kb and 2.4-kb RNAs among the WT virus, del3033–3131, and del3031–3141 with and without a Q87* mutation in the HBx protein. The greater increase of 2.4-kb RNA than 3.5-kb RNA from deletion mutants with impaired HBsAg production could be attributed to additional loss of negative regulatory element(s) for the 2.4-kb RNA. In this regard, Yen and colleagues identified the CCAAT element (nucleotides 3137 to 3141) inside the SPII promoter as a negative regulator of the 2.4-kb RNA (71, 72), while another group found that nucleotides 3160 to 3221 in genotype A (corresponding to nucleotides 3154 to 3215 in genotype C) could inhibit transcription of the 2.4-kb RNA (82).

Paradoxically, for the seven deletion mutants with <1% of the WT level of HBsAg production as 0.6-mer M/S constructs and 0.7-mer M/S/X constructs (del2996–2, del3004–3183, del3016–3144, del3020–3202, del3026–3208, del3004–3174, and del3049–3174), their 0.7-mer L/M/S constructs showed only modest increases in intracellular L protein despite a strong phenotype at the RNA level (Fig. 7B), with a higher level achieved by del3016–3144. This was surprising, considering that L protein secretion was blocked in the absence of S protein coexpression (Fig. 10B and E). The highest levels were rather achieved by del3031–3141 and del3123–3164 with continued (but low) S protein expression (Fig. 7B). For SphI dimers, high intracellular L protein was displayed by these two mutants, a mutant with lost S protein expression (del3016–3144), and del3133–3174. The latter secreted much less HBsAg as a SphI dimer than as a 0.7-mer L/M/S construct (Fig. 2). Of all deletion mutants with impaired HBsAg production, only del3016–3144 and del3031–3141 retained the matrix domain required for L protein interaction with core particles (Fig. 2 and Table 1) (10, 11). Probably, in the absence of S protein coexpression, intracellular L protein is destabilized and degraded unless it can interact with core protein through the matrix domain. Consistent with that hypothesis, we recently found that mutating the S gene ATG codon to prevent expression of full-length S protein not only abolished L protein secretion but also reduced its intracellular level (83). Such a construct continued to produce a small amount of truncated S protein through translation initiation from the next in-frame ATG codon, which was deficient in secretion but could partially sustain intracellular L protein (83).

In the present study, the T156C point mutation to prevent expression of full-length S protein (but not truncated S protein) also reduced intracellular L protein for del3031–3141, especially in HepG2 cells (Fig. 11B and 12B). For the 0.7-mer L/M/S constructs of deletion mutants with complete loss of S protein expression, providing S protein in *trans* only partially increased intracellular levels of L protein even at a 1:1 ratio (Fig. 10D), with modest rescue of L protein secretion (Fig. 10E). Probably intracellular L protein is stabilized much more efficiently by S protein provided in *cis* than in *trans*. L protein retention has been associated with endoplasmic reticulum stress and liver injury (84–89). Overexpression of L protein in transgenic mice can lead to inflammation, regenerative hyperplasia, and liver cancer (90). Mutants such as del3031–3141 and del3016–3144, with elevated intracellular L protein as either SphI dimers or prcccDNA constructs (Fig. 8D and 9D), unabated by the coexistence of extra S protein to mimic the coexistence of WT virus (Fig. 10A and D), should have major clinical implications.

We measured extracellular HBeAg, intracellular core protein, and replicative DNA as products of 3.5-kb pcRNA and pgRNA. Despite increased 3.5-kb RNA for SphI dimers of eight deletion mutants with lost or little HBsAg secretion, the increases in HBeAg were modest and variable among mutants (Fig. 8F). The relative efficiency of HBeAg production among the WT construct, del3031–3141, and del3033–3131 also varied among different experiments in Huh7 cells (Fig. 8F versus Fig. 11E) and between Huh7 and HepG2 cells (Fig. 11E versus Fig. 12E). On the other hand, intracellular core protein levels were elevated for all eight deletion mutants with impaired HBsAg production but neither mutant with sustained HBsAg production (del2910–3092 and del3033–3131) (Fig. 8D). To facilitate data interpretation, it will be helpful in the future to separate pgRNA from pcRNA by a primer extension assay. Besides pgRNA levels, intracellular levels of core protein and replicative DNA could be affected by the efficiency of virion formation/release and the stability of retained core particles (or partially formed virions) if particle release is blocked. In this regard, all eight deletion mutants with impaired HBsAg secretion also showed little virion secretion (Fig. 8C and 9C). The variable length and location of the preS1 deletion would lead to variable deletion in the P protein (Table 1), the enzyme driving HBV genome replication. However, the preS region codes for the nonessential spacer of the P protein, which can be removed without impairing P protein function (91). A previous study found that the 1.1-mer construct of a clinical isolate of genotype C possessed greater replication capacity than a WT clone in transfected Huh7 cells (92). That isolate harbored a 207-nt deletion in the preS1 region (nucleotides 2971 to 3177) in addition to nonsense mutations in the S gene, and putting back the 207 nt reduced genome replication (93). Naturally occurring 5′ preS1 deletion of 15 nt or 18 nt in genotype C would cause an 11-aa deletion at the N terminus of the L protein to become genotype D-like, in addition to a 5-aa or 6-aa deletion in the spacer of the P protein. Interestingly, we found that such deletions, when introduced into 1.1-mer constructs, increased HBV RNA, replicative DNA, and virion production in genotype C clones (60). Therefore, naturally occurring large in-frame deletions in the preS1 region do not necessarily impair P protein function, although that remains to be validated experimentally.

Most deletion mutants with increased intracellular HBx and core proteins (and 3.5-kb RNA for SphI dimers) also showed elevated replicative DNA, whether as SphI dimers or prcccDNA constructs (Fig. 8B and 9B). For both types of construct, del2996–2 showed low replication levels, while del3031–3141 had the highest replication levels. Del3031–3141 was unusual in retaining a low level of intracellular (but not extracellular) S protein and displaying a high level of intracellular L protein and in retaining the matrix domain in its L protein. In Huh7 cells, the X-minus mutant of del3031–3141 continued to manifest higher intracellular replicative DNA than the X-minus mutant of the WT construct (Fig. 11A), suggesting the presence of an HBx-independent mechanism to augment HBV DNA for this mutant in Huh7 cells. The T156C mutation to convert the S gene ATG into ACG markedly reduced replication of this mutant but had limited impact on del3033–3131, which had much greater S protein expression than del3031–3141 to begin with (Fig. 11A). According to our recent study (83), the T156C mutants would very likely generate small amounts of N-terminally truncated S protein for WT virus and del3033–3131 through the second in-frame ATG codon in the S gene (M75). This would be impossible for the del3031–3141 mutant due to the nearly lost SPII promoter. Indeed, Western blot analysis confirmed expression of the truncated S protein (gp19) by the T156C mutants of the WT virus and the del3033–3131 mutant but not the del3031–3141 mutant (Fig. 11B and 12B). Interestingly, preventing the expression of all three envelope proteins through nonsense mutations in both the 5′ preS1 and S regions (T2936A/T216A) significantly increased replicative DNA and core protein for WT virus but not del3031–3141 (Fig. 11A and B). It also increased core protein for del3033–3131, suggesting that blocked virion formation could lead to the retention of core particles and might partly explain the high replication/high core protein phenotypes of del3031–3141. Alternatively, the T216A (nonsense) but not T156C (missense) mutation in the S gene could trigger nonsense-mediated decay of the abundant 2.1-kb RNA for the WT virus and the del3033–3131 mutant to stimulate transcription of the 3.5-kb pgRNA for core protein expression and genome replication. It will be very interesting to determine whether the high replication phenotype of del3031–3141 could be diminished by cotransfection with variable amounts of the S protein construct to rescue virion secretion.

The present study used prcccDNA constructs to further validate findings from SphI dimers, especially in terms of transcriptional regulation among coterminal HBV mRNAs. Most results, including intracellular replicative DNA, extracellular virion DNA, extracellular L/S proteins, and HBsAg, were quite concordant between the two types of constructs. However, del3026–3208 manifested increased intracellular core and HBx proteins as a SphI dimer but not as a prcccDNA (compare Fig. 8D with Fig. 9D), whereas the opposite was true for the del3033–3131 mutant in terms of 3.5-kb RNA and core and HBx proteins. Northern blot analysis revealed a band of slower mobility than the 2.1-/2.4-kb RNA species for some deletion mutants (Fig. 9A, lanes 9 to 12). In this regard, the current prcccDNA system is based on cotransfection of a cloned monomeric HBV genome with the expression construct for Cre recombinase (76). The recombined DNA still contains a 120-nt non-HBV sequence, which is spliced out as an intron during nuclear-cytoplasmic transport. Thus, the need for cotransfection, recombination, and splicing may increase experimental variability and complicate data interpretation. More recently, the minicircle technique allowed production of recombinant cccDNA in Escherichia coli with minimal foreign sequence (94, 95). In at least one version of E. coli-generated cccDNA, the extra 39 nt did not disrupt HBV function (95). It will be of great interest to repeat some of the experiments by direct transfection with such recombinant cccDNA.

In summary, the present study revealed impaired HBsAg production with many naturally occurring 3′ preS1 deletions in the context of the entire HBV genome, mostly through lost transcription of the 2.1-kb RNA, as suggested by data from 0.7-mer M/S/X constructs, but probably some through the additional mechanism of a greatly increased L/S protein ratio. Lost HBsAg production at the transcriptional level was accompanied by increased 3.5-kb, 0.7-kb, and especially 2.4-kb RNAs but for the 3.5-kb and 2.4-kb RNAs not necessarily through lost transcriptional interference from 2.1-kb RNA. At the protein level, most deletion mutants with lost HBsAg production manifested higher intracellular HBx and core proteins, although the highest L protein levels were achieved by mutants with no loss of matrix domain or residual S protein expression. Increased intracellular replicative DNA for such mutants could be attributed to upregulation of HBx protein expression in addition to lost virion secretion. Overall, the 3′ preS1 deletions are similar to CPMs in upregulating HBV genome replication, although transcription of the 2.4-kb RNA for L protein and the 0.7-kb RNA for HBx protein would be enhanced by 3′ preS1 deletions but diminished by CPMs (64). In this regard, HBx protein has been directly linked to hepatocarcinogenesis, although many studies on HBx protein were considered artificial due to its overproduction by a strong exogenous promoter (9). Our results suggest that naturally occurring preS1 deletions could upregulate HBx protein expression, thus making some of the previous studies relevant for patients infected with such mutants. Amino acid substitutions in the HBx protein secondary to CPMs might render the HBx protein more oncogenic (27). Whether naturally occurring 3′ preS1 deletions synergize with CPMs to promote hepatocarcinogenesis warrants further investigation.

## MATERIALS AND METHODS

### Full-length HBV replication constructs and subgenomic expression constructs for envelope proteins.

Geno27.2 of genotype C (subgenotype C2) (GenBank accession number KU964186) has been characterized (65, 66). It has an intact genome of 3,215 nt and contains WT precore and core promoter sequences. The SphI dimer had two copies of the HBV genome inserted via the unique SphI site in the pUC18 vector. The P-minus mutant had a mutated (from ATG to ACG) translation initiation site to prevent expression of full-length P protein as required for genome replication. The prcccDNA was produced from a precursor plasmid containing a floxed HBV monomeric genome with an exogenous sequence of 2,195 nt inserted between nucleotides 202 and 203 in the S region (76). Its cotransfection with the expression construct for Cre recombinase would lead to Cre/*loxP*-mediated DNA excision, and the remaining 120 nt of non-HBV sequence from the primary transcripts would be spliced out as an intron during nuclear-cytoplasmic transport (76). Subgenomic constructs were employed to express all three envelope proteins or just M and S proteins, with or without simultaneous HBx protein expression. We previously made a 0.7-mer L/M/S expression construct (17). It had nucleotides 2694 to 3221 and 1 to 835 of a genotype A clone (with an added SacI site at its 5′ end and a HindIII site at its 3′ end) inserted between the SacI and HindIII sites of the pBluescript SK(−) vector, followed by the HBV posttranscriptional regulatory element (nucleotides 970 to 1770) fused with a 300-bp SV40 polyadenylation site inserted between the HindIII and XhoI sites (Fig. 1C). This allowed highly efficient HBsAg production (Fig. 3). In the present study, replacing the SacI-HindIII fragment of genotype A with nucleotides 2688 to 3215 and 1 to 835 or nucleotides 2954 to 3215 and 1 to 835 of geno27.2 generated the 0.7-mer L/M/S expression construct or the 0.6-mer M/S construct, respectively, of genotype C. Another 0.7-mer construct capable of expressing M, S, and HBx protein was created by inserting nucleotides 2688 to 3215 and 1 to 1960 of geno27.2 into the SacI and HindIII sites, respectively, of the pBluescript SK(−) vector (Fig. 1C).

### The preS deletion and site-directed mutants.

The preS deletions were introduced into geno27.2 by PCR using high-fidelity DNA polymerase (NEB), followed by restriction fragment (ApaI-AvrII) exchange with the SphI dimer. Since enzymatic digestion would convert a dimer into a monomer, the monomeric forms of the deletion mutants were reconverted into SphI dimers using two-way molecular ligation (96). The deletions were also introduced into the subgenomic expression constructs of envelope proteins. To serve as a negative control for envelope protein expression, a 1.1-mer construct of another genotype C clone, geno17.3 (65), was rendered L-minus and S-minus by the G2858A and G261A nonsense mutations in the preS1 and S regions, respectively (66). The L-minus mutant and S-minus mutant of the SphI dimer were generated by the T2936A nonsense mutation of preS1 codon 30 (L30*) and the T156C mutation to convert the ATG initiation codon of the S gene into ACG, respectively. The envelope-null mutant has the T2936A nonsense mutation in the preS1 region in addition to the T216A nonsense mutation at codon 21 of the S gene (L21*). The X-minus mutant has a C1632T nonsense mutation at codon 87 of the X gene (Q87*). Mutations were introduced by overlap-extension PCR, and the resultant SphI monomer was reconverted into a SphI dimer using our established protocol (96).

### Transient transfection and protein analysis.

Huh7 cells were cultured in Dulbecco^’^s modified Eagle medium (Gibco) supplemented with 10% fetal bovine serum (Gibco). HepG2 cells were cultured in minimum essential medium (Gibco) supplemented with 10% fetal bovine serum (Gibco). Transient transfection was performed on cells seeded in six-well plates at a density of 80 to 90%, using the Lipofectamine 3000 transfection kit (Invitrogen) and 2 μg plasmid DNA. For prcccDNA, it was cotransfected with CMV-*cre* (expression construct for Cre recombinase) at a 1:1 ratio. Cells and culture supernatant were harvested 4 days posttransfection with subgenomic constructs and 5 days posttransfection with SphI dimer or prcccDNA. Cells were lysed in 100 μl of lysis buffer (10 mM HEPES [pH 7.5], 100 mM NaCl, 1 mM EDTA, 1% NP-40). A 1/10th volume of the cell lysate was separated by SDS-PAGE and transferred to a polyvinylidene difluoride (PVDF) membrane. The membrane was blocked at room temperature for 1 h with 5% milk dissolved in Tris-buffered saline with 0.1% Tween 20 (TBST) buffer, followed by incubation at 4°C overnight with a 1:1,000 dilution of 7H11, an anti-preS1 mouse MAb (97). After further incubation with horseradish peroxidase (HRP)-conjugated goat anti-mouse IgG antibody (1:10,000 dilution), signals were revealed by the enhanced chemiluminescence reagent Plus (PerkinElmer). For an internal control, the membrane was incubated with a mouse anti-glyceraldehyde-3-phosphate dehydrogenase (GAPDH) antibody (1:5,000 dilution) followed by HRP-conjugated goat anti-mouse IgG antibody (1:20,000 dilution). S protein was detected by a rabbit polyclonal anti-HBs antibody (Novus) at 1:3,000 dilution followed by HRP-conjugated goat anti-rabbit IgG antibody at 1:10,000 dilution. Core protein was detected by MAb 2A7 at 1:1,000 dilution followed by HRP-conjugated goat anti-mouse IgG antibody at 1:3,000 dilution. The MAb targeted residues 141 to 154 in core protein (98). HBx was detected by MAb 20F3 at 1:2,000 dilution and HRP-conjugated goat anti-mouse IgG antibody at 1:2,000 dilution (99). 20F3 was generated by immunization of mice with the full-length HBx protein of subgenotype A2 expressed in E. coli, and it displayed efficient detection of HBx protein from genotypes A, B, and C, with weaker detection of HBx protein from genotype D. Its epitope has been mapped to amino acids 102 to 119 in HBx protein.

To detect secreted envelope proteins by Western blotting, 600 μl of culture supernatant was mixed with 200 μl of 36% polyethylene glycol (PEG) 8000 solution in phosphate-buffered saline (PBS). After rotation at 4°C overnight, the samples were centrifuged at 12,000 rpm for 40 min, and the pellet was resuspended in 30 μl TN buffer (10 mM Tris-HCl [pH 8.0], 150 mM NaCl) for SDS-PAGE. Secreted HBsAg and HBeAg were quantified by commercial ELISA kits (KHB, Shanghai, China) according to the manufacturer’s instructions. Samples were properly diluted to reach optical density at 450 nm (OD_450_) values of around 1.0 to 2.0.

### Southern blot analysis of replicative HBV DNA and virion DNA.

The details of HBV core particle extraction have been described (17, 26). In brief, one-half of the cell lysate from 6-well plates was incubated at 37°C for 15 min with 0.5 U DNase I and 7.5 U mung bean nuclease. Core particles was precipitated with PEG 8000 and further treated with DNase I and mung bean nuclease, followed by proteinase K digestion, DNA extraction with Tris-saturated phenol, and precipitation with ethanol in the presence of glycogen. Extracted DNA was separated in a 1.3% agarose gel. After denaturation and renaturation, DNA in the gel was transferred to a nylon membrane (Roche). The membrane was hybridized with a ^32^P-labeled full-length HBV DNA probe at 42°C overnight and washed at 55°C successively in 2× SSC (1× SSC is 0.15 M NaCl plus 0.015 M sodium citrate)/0.1% SDS and 0.5× SSC/0.1% SDS solutions. The signals were revealed by Typhoon software. The 3.2-kb HBV DNA for probe production was obtained by nested PCR amplification of nucleotides 1828 to 3215 and 1 to 1823 from cloned geno27.2 and was labeled with [α-^32^P]dCTP using the random primed DNA labeling kit (Roche). Virions were immunoprecipitated from 1.4 ml of precleared culture supernatant at day 5 posttransfection by a combination of 1 μl rabbit polyclonal anti-HBs antibody (Novus) and 3 μl rabbit polyclonal anti-preS1 antibody against preS1 residues 12 to 46 of genotypes B and C (GenScript) preconjugated to 10 μl protein G-agarose beads (69), followed by DNase I/mung bean nuclease treatment, protease K digestion, and DNA extraction (17, 33, 65, 66). Virion DNA was subjected to Southern blot analysis in the same way as intracellular replicative DNA.

### RNA extraction and Northern blot analysis.

Huh 7 cells were lysed by TRI reagent (MRC) at day 3 posttransfection, and RNA was extracted as described previously (64, 66, 100). Briefly, the samples were mixed with chloroform at room temperature for 5 min. RNA was precipitated by isopropanol, followed by sequential washes with 75% ethanol and absolute ethanol. The total RNA (10 μg) in 5× RNA loading buffer (TaKaRa) was denatured at 65°C for 10 min and separated in a 1.3% agarose gel with morpholinepropanesulfonic acid (MOPS) and formaldehyde. The gel was soaked in 0.05 N NaOH solution, and RNA was transferred to a nylon membrane (Roche). The Northern blot was hybridized with [α-^32^P]dCTP-labeled HBV DNA probe prepared from the full-length HBV genome just like for Southern blotting or a 0.7-kb HBV DNA obtained by nested PCR amplification of nucleotides 1266 and 1949 from cloned geno27.2. The remaining operations were the same as for Southern blotting, although diethyl pyrocarbonate (DEPC)-treated reagents were used. We found that use of the full-length probe increased sensitivity for Southern blotting but made it difficult to visualize the 0.7-kb RNA in Northern blotting (compare Fig. 3A with Fig. 5A and 7A).
